# Dynamic arterial elastance as a predictor of arterial pressure response to norepinephrine weaning in mechanically ventilated patients with vasoplegic syndrome—a systematic review and meta-analysis

**DOI:** 10.3389/fcvm.2024.1350847

**Published:** 2024-02-08

**Authors:** Xiaoyang Zhou, Caibao Hu, Jianneng Pan, Chang Xu, Zhaojun Xu, Tao Pan, Bixin Chen

**Affiliations:** ^1^ Department of Intensive Care Medicine, Ningbo No.2 Hospital, Ningbo, Zhejiang, China; ^2^ Department of Intensive Care Medicine, Affiliated Zhejiang Hospital, Zhejiang University School of Medicine, Hangzhou, Zhejiang, China

**Keywords:** dynamic arterial elastance, arterial tone, hypotension, norepinephrine, shock

## Abstract

**Introduction:**

During the de-escalation phase of circulatory shock, norepinephrine weaning may induce diverse arterial pressure responses in patients with different vasomotor tones. Dynamic arterial elastance (Ea_dyn_) has been extensively studied to predict the arterial pressure response to interventions. We conducted this meta-analysis to systematically assess the predictive performance of Ea_dyn_ for the mean arterial pressure (MAP) response to norepinephrine weaning in mechanically ventilated patients with vasoplegic syndrome.

**Materials and methods:**

A systematic literature search was conducted on May 29, 2023 (updated on January 21, 2024), to identify relevant studies from electronic databases. The area under the hierarchical summary receiver operating characteristic curve (AUHSROC) was estimated as the primary measure of diagnostic accuracy because of the varied thresholds reported. Additionally, we observed the distribution of the cutoff values of Ea_dyn_, while computing the optimal value and its corresponding 95% confidential interval (CI).

**Results:**

A total of 5 prospective studies met eligibility, comprising 183 participants, of whom 67 (37%) were MAP responders. Ea_dyn_ possessed an excellent ability to predict the MAP response to norepinephrine weaning in patients with vasoplegic syndrome, with an AUHSROC of 0.93 (95% CI: 0.91–0.95), a pooled sensitivity of 0.94 (95% CI: 0.85–0.98), a pooled specificity of 0.73 (95% CI: 0.65–0.81), and a pooled diagnostic odds ratio of 32.4 (95% CI: 11.7–89.9). The cutoff values of Ea_dyn_ presented a nearly conically symmetrical distribution; the mean and median cutoff values were 0.89 (95% CI: 0.80–0.98) and 0.90 (95% CI: not estimable), respectively.

**Conclusions:**

This meta-analysis with limited evidences demonstrates that Ea_dyn_ may be a reliable predictor of the MAP response to norepinephrine weaning in mechanically ventilated patients with vasoplegic syndrome.

**Systematic Review Registration:**

PROSPERO CRD42023430362.

## Introduction

Currently, norepinephrine is recommended as the first-line vasopressor for the treatment of circulatory shock (1, 2). Norepinephrine can effectively maintain targeted arterial pressure primarily by restoring vasomotor tone through stimulating *α*_1_-adrenergic receptors (3). Clinically, the de-escalation of norepinephrine therapy should be considered early to avoid tissue hypoperfusion associated with excessive vasoconstriction once resolving the cause of circulatory shock and achieving hemodynamic stability. However, decreasing the norepinephrine dosage (norepinephrine weaning) may induce diverse arterial pressure responses in patients with different vasomotor tones due to its potent *α*_1_-adrenergic properties. For instance, early weaning from norepinephrine infusion may cause unnecessary exposure to arterial hypotension in those patients with persistent depressed vasomotor tone. Inversely, prolonged norepinephrine infusion may induce excessive vasoconstriction in those with restored vascular tone (4). Therefore, the evaluation of vasomotor tone may be helpful to discriminate against those patients who will successfully wean from norepinephrine infusion and those who will fail.

Historically, dynamic arterial elastance (Ea_dyn_), defined by the ratio of pulse pressure variation (PPV) to stroke volume variation (SVV), has been extensively described as a functional measure of arterial tone (5, 6). According to the calculation formula, Ea_dyn_ defines the changes in the arterial pulse pressure caused by the changes in left ventricular stroke volume (SV) related to the intrathoracic pressure changes during a respiratory cycle (7, 8). Furthermore, the term “elastance” indicates that Ea_dyn_ is related to arterial stiffness, which is partially determined by vasomotor tone (7, 9). Accordingly, Ea_dyn_ describes the dynamic interaction between changes in pressure and flow and evaluates the dynamical changes in arterial tone (8). Over the past decade, Ea_dyn_ has been demonstrated as a reliable predictor of arterial pressure changes related to fluid challenge (8, 10, 11). However, the predictive performance of Ea_dyn_ for arterial pressure response to norepinephrine weaning has not been systematically evaluated, even though the relationship between Ea_dyn_ and arterial pressure changes induced by norepinephrine weaning has been widely investigated (12, 13). Therefore, we conducted this meta-analysis to assess the diagnostic accuracy of Ea_dyn_ for predicting the mean arterial pressure (MAP) response to norepinephrine weaning.

## Materials and methods

This systematic meta-analysis was carried out following the guidelines of the Preferred Reporting Items for a Systematic Review and Meta-analysis of Diagnostic Test Accuracy (14). The study protocol was registered in advance at the international prospective register of systematic reviews (PROSPERO, CRD42023430362) before initiating the study.

### Data sources and search strategy

On May 29, 2023, two reviewers (XZ and CX) independently performed a systematic literature search in the PubMed, Web of Science, Embase, and Cochrane Library to identify studies that investigated the diagnostic accuracy of Ea_dyn_ for predicting the MAP response to norepinephrine weaning in mechanically ventilated patients with vasoplegic syndrome, without any restriction on the published date or language. Furthermore, they also reviewed the bibliographies of previous publications to identify relevant studies. The same 2 reviewers (XZ and CX) updated the literature search on January 21, 2024. We presented the search strategies in detail in Supplementary Table S1 (see Additional file 1).

### Eligibility criteria

Candidate studies were deemed eligible if they met all of the following criteria: (1) prospective or retrospective studies on mechanically ventilated adults with vasoplegic syndrome (age >18 years) who received norepinephrine to maintain arterial pressure and whose physicians in charge decided to decrease the norepinephrine dose; (2) the MAP changes after decreasing the norepinephrine dose were considered as the reference gold standard to define MAP responsiveness, regardless of the threshold value; (3) the Ea_dyn_ at baseline (before norepinephrine weaning) was measured as the index test; and (4) reporting sufficient information to construct a 2 × 2 contingency table. Vasoplegic syndrome was predefined as persistent arterial hypotension with normal or high cardiac output and low systemic vascular resistance, despite adequate fluid resuscitation (15). We excluded those studies that met anyone of the following criteria: (1) enrolling patients with spontaneous breathing or patients who did not meet the definition of vasoplegic syndrome; (2) lacking sufficient information on the diagnostic accuracy of Ea_dyn_; or (3) conference abstracts without a full text.

### Study selection and data extraction

Two authors (JP and TP) initially eliminated duplicates from the searched records. After deduplication, they independently reviewed the titles and abstracts of the remaining records. Subsequently, the full texts of the candidate studies were carefully reviewed by the same two independent authors to determine whether they met the eligibility or not. A discussion was required to resolve the disagreements between the two authors; if necessary, a third reviewer (ZX) was involved. Supplementary Table S2 (see Additional file 1) presented the reasons for precluding the ineligible studies.

Regarding the data extraction, the same two authors (JP and TP) independently utilized a pre-customized extraction form to extract the information of interest from the included studies, including the study characteristics, patient characteristics, and the diagnostic accuracy measures of Ea_dyn_. To facilitate constructing a 2 × 2 contingency table, we computed the true positive, false positive, false negative, and true negative events based on the sensitivity, specificity, and sample size in each included study. If the needed information is not reported, we would return to the receiver operating characteristic (ROC) curve to estimate the sensitivity and specificity. If any disagreement exists, they would jointly review the full text to reach a consensus.

### Quality assessment

Another two authors (BC and CH) assessed the methodological quality of each included study independently using the Quality Assessment of Diagnostic Accuracy Studies (QUADAS)-2 tool (16). The QUADAS-2 consists of 4 domains that were assessed for the risk of bias: patient selection, index test, reference standard, and flow and timing; the first 3 domains were also assessed for applicability concerns. If there is any disagreement, the third reviewer (ZX) would join the discussion to help conclude.

### Statistical analysis

In a preliminary analysis, we presented the derived estimates of sensitivity and specificity on the forest plot and the ROC space to evaluate the between-study variations in the diagnostic accuracy of Ea_dyn_. Additionally, the between-study heterogeneities were also assessed by calculating Cochran's Q test and I^2^ statistics. Data syntheses were then performed within the random-effect bivariate model to calculate the pooled sensitivity, pooled specificity and pooled diagnostic odds ratio (DOR) (17, 18). The bivariate model allows for the expected trade-off in sensitivity and specificity due to the between-study variations in the thresholds of Ea_dyn_ (17). Given the expected threshold effect, which was assessed statistically by calculating the Spearman correlation coefficient between the logit of sensitivity and the logit of 1-specificity (19), we adopted a hierarchical regression model to fit the hierarchical summary receiver operating characteristic (HSROC) curve (20). Considering that the summary point of sensitivity and specificity represents an estimate of a notional unspecified average of mixed thresholds that cannot be clinically interpreted, we reported the area under the HSROC curve (AUHSROC) as the main measure of diagnostic accuracy (17). Stata/SE 15.0 software integrating the MIDAS and METANDI modules (Stata-Corp, College Station, TX, USA) was used to perform statistical analysis. A two-tailed *P* < 0.05 was considered statistically significant.

To facilitate clinical decision-making, we estimated the optimal threshold of Ea_dyn_ along with its corresponding 95% confidential interval (CI) by computing the mean and median cutoff values and observing the distribution, dispersion, and central tendency of the reported cutoff values. Meanwhile, we constructed a Bayesian nomogram to calculate the post-test probability to facilitate a better interpretation of the findings. In addition, a sensitivity analysis was conducted to assess the robustness of the results by excluding the study introducing a high risk of bias. If sufficient studies were identified, we would assess the publication bias by using Deeks’ funnel plot asymmetry test (21), and we would also conduct subgroup analyses based on the shock type (septic or postoperative) and the technique measuring SVV.

## Results

**Figure 1:**
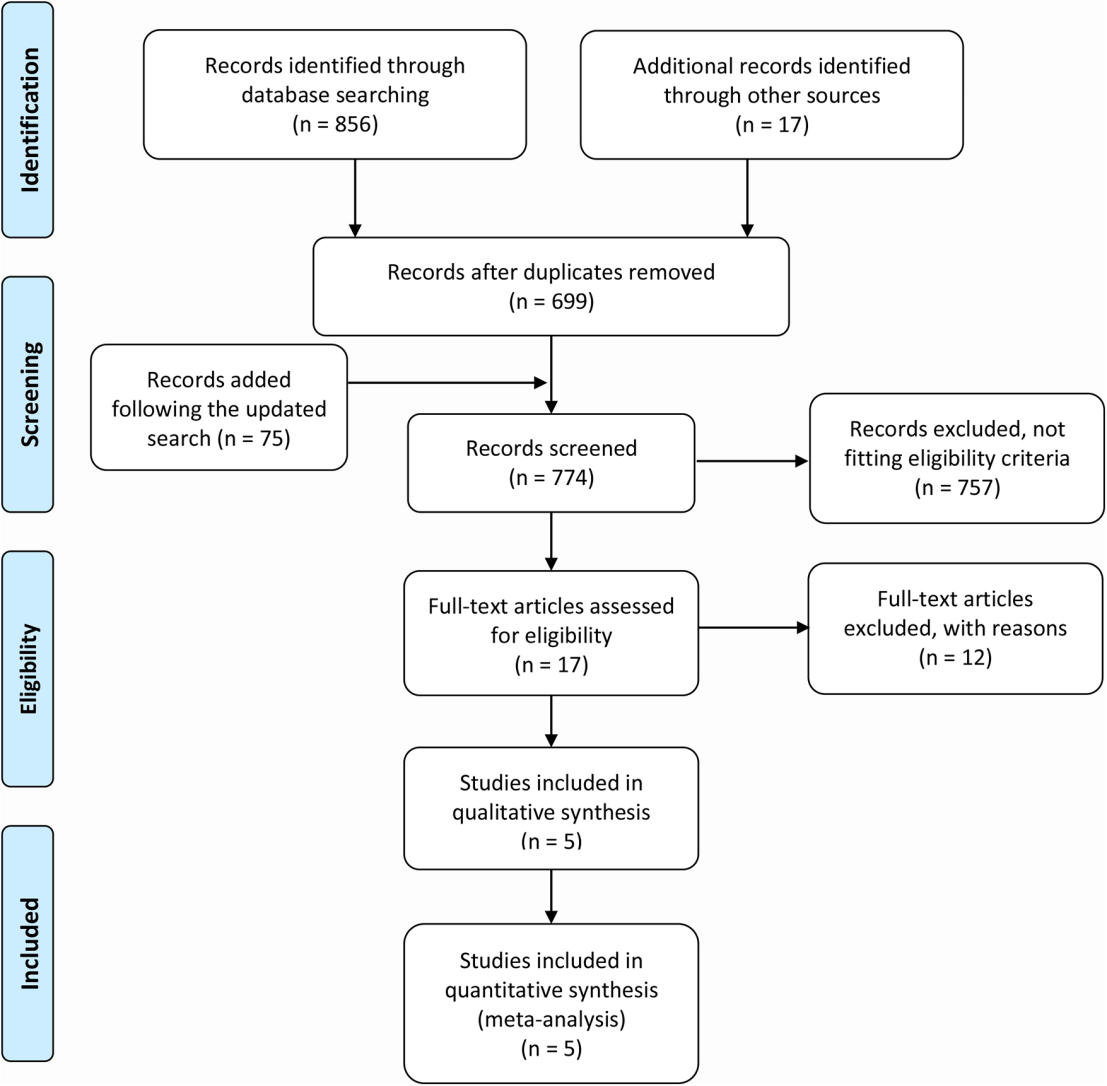
The flowchart of study selection.

Figure 1 depicts the flowchart of study selection. We identified a total of 856 records from the database search and retrieved additional 17 records from other publications, and additional 75 citations were added following the updated search. After precluding 174 duplicates and 769 irrelevant records, 5 studies (4, 12, 13, 22, 23) that met the eligibility were finally included in the quantitative analysis.

### Baseline characteristics and quality assessment

All the included studies were prospective designs and conducted in the intensive care unit (ICU). A total of 183 patients were enrolled, with sample sizes ranging from 32 to 42. The infused dose of norepinephrine at baseline ranged from 0.14 to 0.47 µg kg^−1^ min^−1^. The subjects in 3 studies (4, 13, 22) were patients with septic shock and in the remaining 2 studies (12, 23) were the mixed population (including surgical patients and septic patients). Three studies (12, 13, 23) used the threshold value of 10% to define MAP responsiveness, and 2 studies (4, 22) defined MAP responsiveness using the threshold value of 15%. The SVV was measured by using arterial waveform analysis in 2 studies (12, 13), using the pulse indicator continuous cardiac output technique in 2 studies (4, 22), and using transthoracic echocardiography in the remaining 1 study (23). Table 1 describes the detailed study and patient characteristics, and Supplementary Table S3 (see Additional file 1) records detailed diagnostic accuracies of Ea_dyn_ from each included study.

**Table 1:** Baseline characteristics.

Study No.	Author/year	Design, setting, and location	Subjects	Sample size	Age (years)	Tide volume (mL kg^−1^)	PEEP (cm H_2_O)	CO (L min^−1^)	MAP (mean or median, mm Hg)	No. of MAP responder (n, %)	NE dose at baseline (µg kg^−1^ min^−1^)	NE dose for one reduction	MAP responsiveness	Technique measuring SVV
1	Guinot/2015	Prospective study; ICU; France	Septic shock patients under mechanical ventilation	35	NR	7.7	7	6.1	78	13 (37)	0.33	3.3 µg min^−1^	≥15% decrease	PICCO
2	Liang/2017	Prospective study; ICU; China	Septic shock patients under mechanical ventilation	32	59	7.3	6.1	6.1	79	13 (41)	0.47	3.3 µg min^−1^	≥15% decrease	PICCO
3	Bar/2018	Prospective study; ICU; France	Mechanically ventilated patients with vasoplegic syndrome (cardiac postoperative 40%, septic shock 40%)	35	65	NR	NR	4.4	80	11 (31)	0.16	3.3 µg min^−1^	≥10% decrease	Arterial waveform analysis
4	Nguyen/2021	Prospective study; ICU; France	Mechanically ventilated patients with vasoplegic syndrome (postoperative 41%, septic shock 41%)	39	67	NR	NR	4.9	79	12 (31)	0.2	3.3 µg min^−1^	≥10% decrease	TTE
5	Persona/2022	Prospective study; ICU; Italy	Septic shock patients under mechanical ventilation	42	57	7.5	NR	4.4	76	18 (43)	0.14	0.03 µg kg^−1^ min^−1^	≥10% decrease	Arterial waveform analysis

Data on the age, tide volume, PEEP, CO, MAP, and NE dose at baseline are presented as mean or median values. No. number; NE norepinephrine; PEEP positive end-expiratory pressure; CO cardiac output; MAP mean arterial pressure; ICU intensive care unit; SVV, stroke volume variation; NR, no record; PICCO pulse indicator continuous cardiac output; TTE transthoracic echocardiography.

As shown in Table 2, none of the included studies was judged as having high methodological quality; most included studies were judged as unclear risk of bias in the domains of patient selection and index test due to lacking sufficient information to support these judgments. It is noteworthy that the study by Persona et al. (13) introduced a high risk of bias in the domain of reference standard because the norepinephrine dose for one reduction was lower than that in the other studies (Table 1), which might potentially decrease the proportion of MAP responder.

**Table 2:** Methodological quality of each included study.

Study	Risk of bias				Applicability concerns		
	Patient selection	Index test	Reference standard	Flow and timing	Patient selection	Index test	Reference standard
Guinot/2015	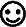	?	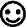	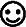	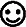	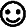	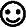
Liang/2017	?	?	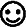	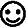	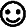	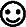	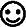
Bar/2018	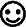	?	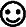	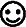	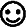	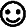	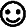
Nguyen/2021	?	?	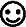	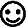	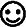	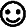	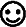
Persona/2022	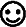	?	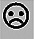	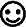	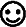	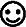	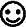

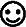
 low risk; 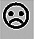 high risk;? unclear risk.

### Diagnostic accuracy of dynamic arterial elastance

**Figure 2:**
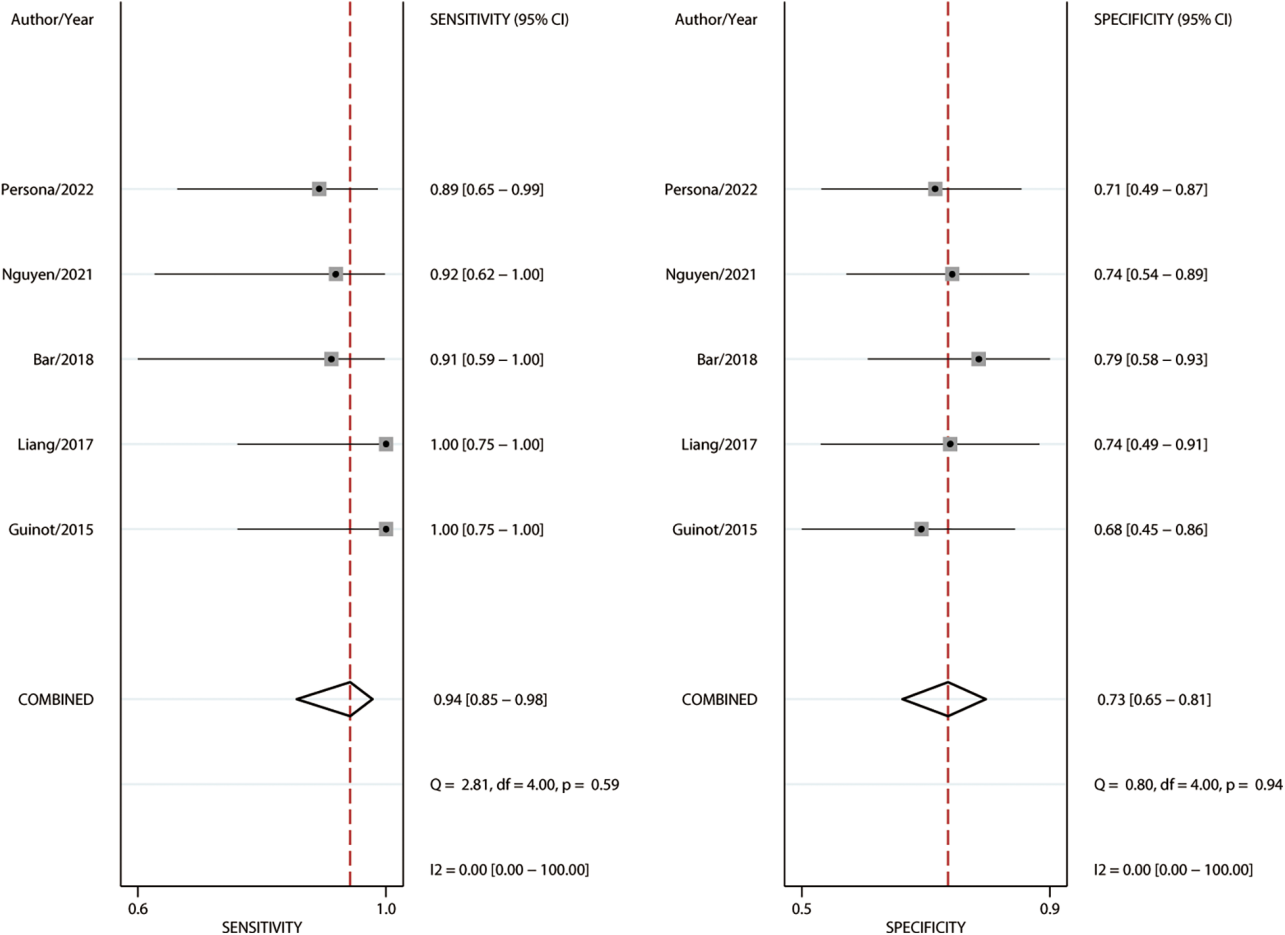
Forest plot of sensitivity and specificity for Ea_dyn_ to predict the MAP response to norepinephrine weaning. Ea_dyn_, dynamic arterial elastance; MAP, mean arterial pressure.

**Figure 3:**
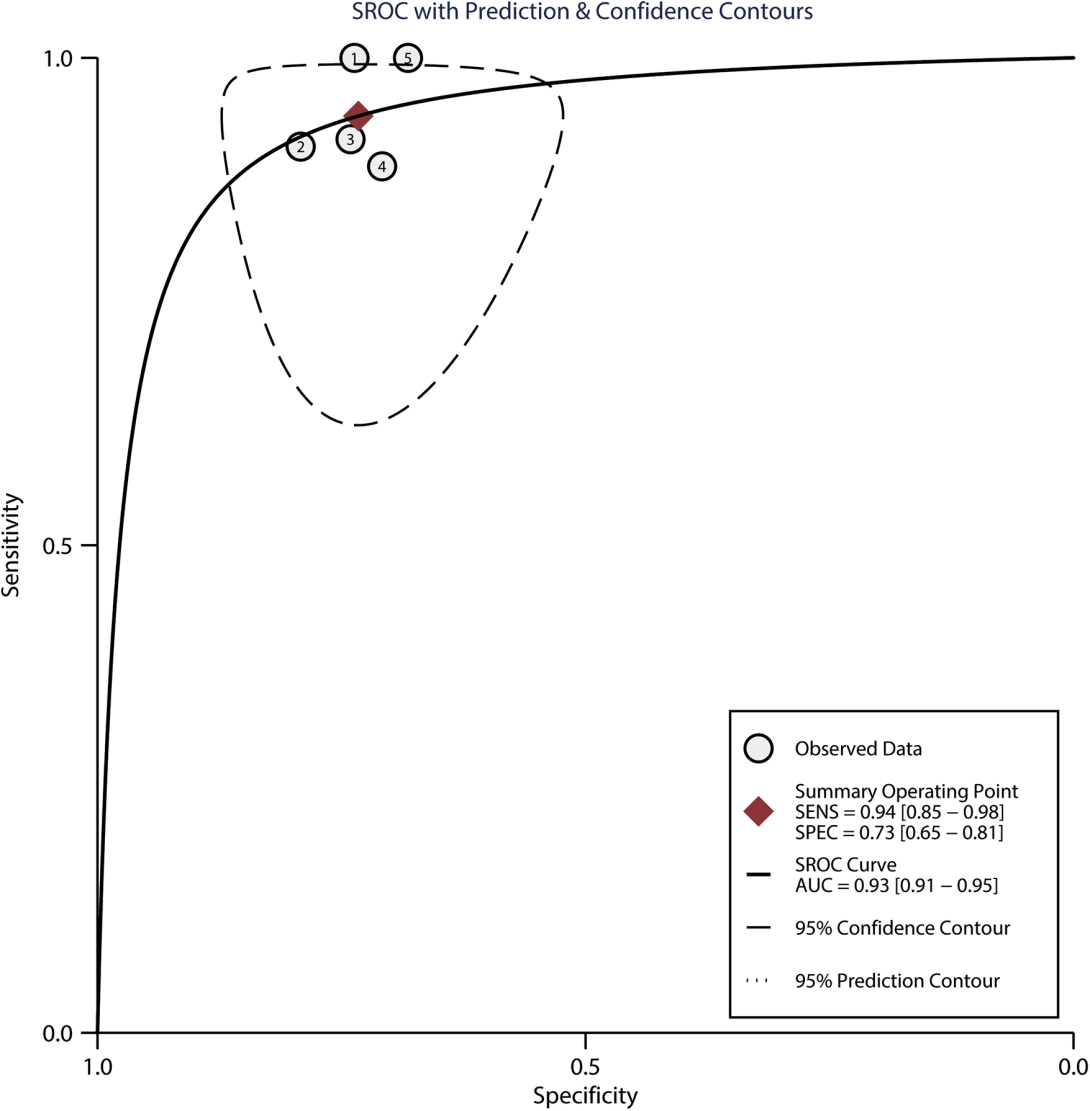
HSROC curve for Ea_dyn_ to predict the MAP response to norepinephrine weaning. The summary point (maroon solid square) with its corresponding 95% confidence region (black dashed line) represents the average sensitivity and specificity estimate. HSROC, hierarchical summary receiver operating characteristic; Ea_dyn_, dynamic arterial elastance; MAP, mean arterial pressure; SEN, sensitivity; SPEC, specificity; AUC, area under the curve.

**Figure 4:**
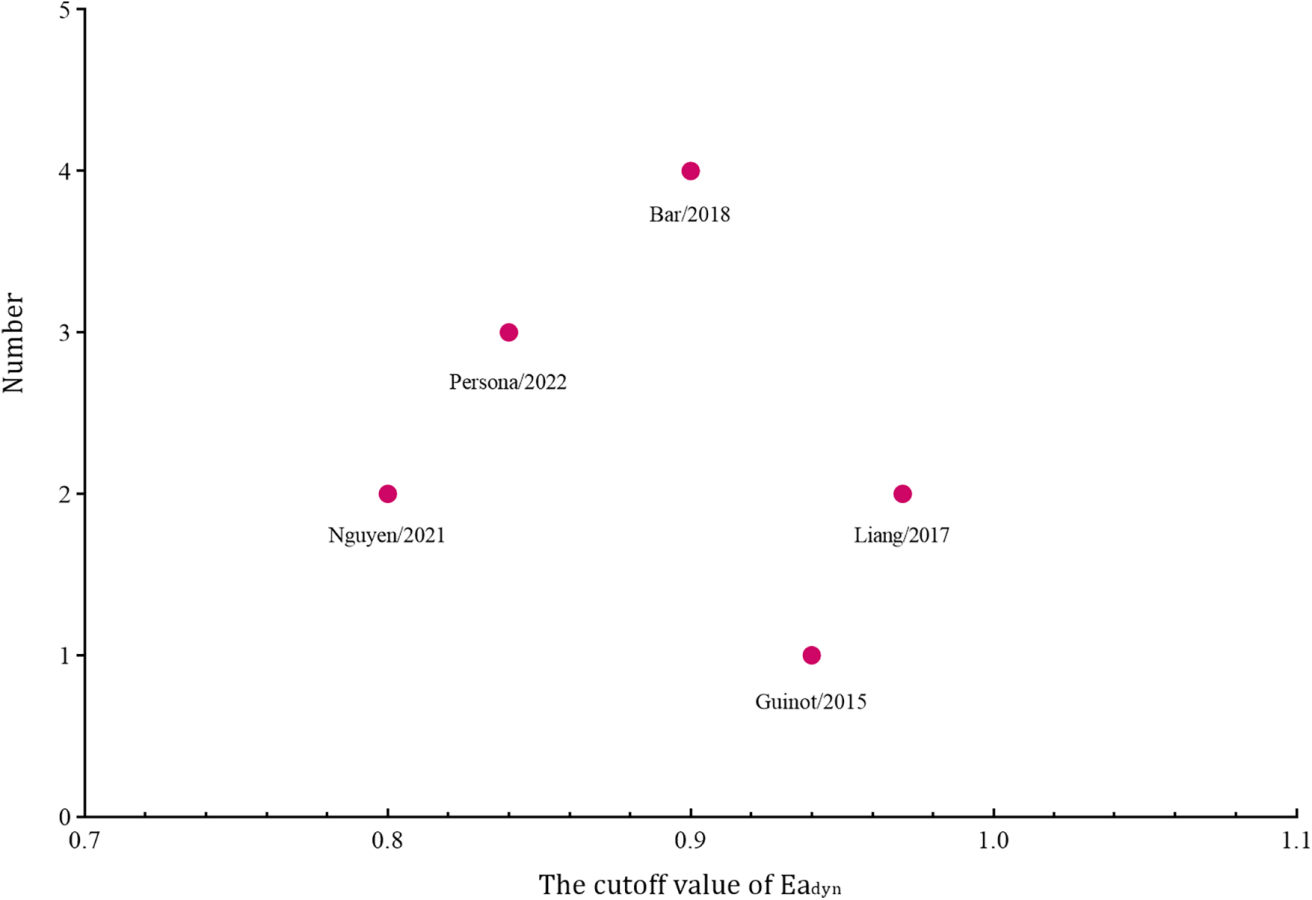
Scatter plot of the cutoff values of Ea_dyn_. Ea_dyn_, dynamic arterial elastance.

**Figure 5:**
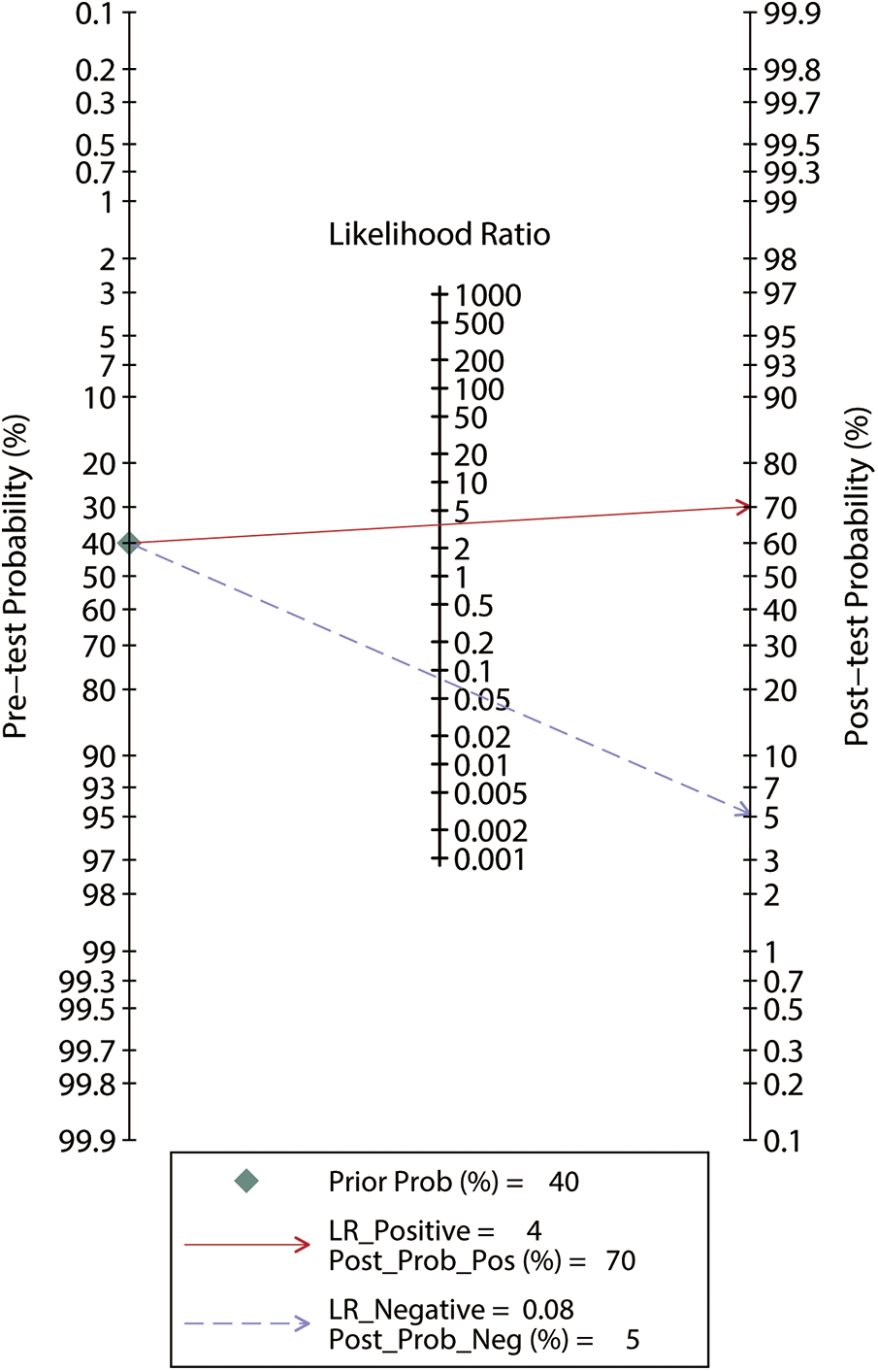
Bayes nomogram for Ea_dyn_ to predict the MAP response to norepinephrine weaning. If an average-risk population has an assumed pretest probability of fluid responder of 40%, the probability of MAP responder will increase to 70% when the test is positive and decrease to 5% when the test is negative. Ea_dyn_, dynamic arterial elastance; MAP, mean arterial pressure.

Among the 183 enrolled participants, 67 (37%) were MAP responders who manifested a significant decrease in MAP after reducing the norepinephrine dosage. We observed no heterogeneity in the sensitivity and specificity across the included studies (Figure 2). The pooled results indicated that Ea_dyn_ was a reliable predictor of the MAP response to norepinephrine weaning in mechanically ventilated patients with vasoplegic syndrome, with an AUHSROC of 0.93 (95% CI: 0.91–0.95), a pooled sensitivity of 0.94 (95% CI: 0.85–0.98), a pooled specificity of 0.73 (95% CI: 0.65–0.81), and a pooled DOR of 32.4 (95% CI: 11.7–89.9) (Figure 3). All of the included studies recorded a cutoff value of Ea_dyn_, ranging from 0.80 to 0.97. The scatter plot of the cutoff values of Ea_dyn_ presented a nearly conically symmetrical distribution (Figure 4); the mean and median cutoff values were 0.89 (95% CI: 0.80–0.98) and 0.90 (95% CI: not estimable), respectively. Thus, the range of 0.80–0.98 may represent the optimal 95% CI for predicting the MAP response to norepinephrine weaning. Consequently, according to the Bayes nomogram (Figure 5), if an average-risk population has an assumed pretest probability of MAP responder of 40% (as estimated in this meta-analysis), the probability of MAP responder will increase to 70% when the measured Ea_dyn_ is greater than 0.98 and decrease to 5% when the measured Ea_dyn_ is less than 0.80.

### Sensitivity analysis

Since the study by Persona et al. (13) introduced a high risk of bias in the domain of reference standard, it was excluded from the sensitivity analysis. Compared with the primary analysis, the sensitivity analysis indicated a comparable diagnostic accuracy of Ea_dyn_, with an AUHSROC of 0.95 (95% CI: 0.93–0.97), a pooled sensitivity of 96% (95% CI: 85%–99%), a pooled specificity of 74% (95% CI: 64%–82%), and a pooled DOR of 42.9 (95% CI: 12.1–152.6), confirming the robustness of our findings. Due to the limited included studies, we abandoned the scheduled plans of evaluating the publication bias and conducting subgroup analyses based on the shock type and the technique measuring SVV.

## Discussion

This systematic meta-analysis of 5 observational studies sought to assess the diagnostic accuracy of Ea_dyn_ in the prediction of arterial pressure response to norepinephrine weaning. Despite providing limited evidences, the principal findings indicate that Ea_dyn_ may be a reliable predictor of the MAP response to norepinephrine weaning in mechanically ventilated patients with vasoplegic syndrome, and the range of 0.80–0.98 may represent the optimal 95% CI for the prediction, within which the measured Ea_dyn_ could not reliably predict the MAP response to norepinephrine weaning.

The current findings provide an important clinical implication that the measurement of Ea_dyn_ before decreasing the norepinephrine dosage may be helpful to recognize which one will experience an arterial pressure reduction associated with norepinephrine weaning. Before the index can be applied widely in clinical practice, some physiological rationales should be acknowledged. As arterial pressure is generated by the interaction of the arterial tone with the blood flow, the magnitude of arterial pulse pressure can theoretically reflect the SV if the heart rate and arterial tone keep unchanged. In general situations, the variations in pulse pressure (i.e., PPV) should be synchronized with the changes in SV induced by intrathoracic pressure changes (i.e., SVV) because the variations in heart rate and arterial tone during a respiratory cycle are typically negligible (24). In the case of circulatory shock, however, vasopressors will exhibit a substantial impact on the arterial tone and, inevitably, affect the interaction between PPV and SVV (25, 26). In this case, the interrelation between PPV and SVV will present a nonlinear relationship, and the ratio of PPV to SVV (i.e., Ea_dyn_) could functionally describe the dynamical changes in arterial tone and dynamically depict the instantaneous arterial pressure-flow relationship, in analogy to defining the cardiac function curve by preload-responsiveness variables (6, 25). Our meta-analysis together with a previous study (8) demonstrated an excellent ability of Ea_dyn_ to predict the changes in arterial pressure related to treatment adjustment (including fluid expansion and norepinephrine weaning). Similarly, Ea_dyn_ was also recently documented as an adjustable predictor of post-induction hypotension in patients undergoing general anesthesia (27).

Instead of representing a true surrogate of arterial load, Ea_dyn_ is actually an index that informs about the balance between blood flow and arterial load (i.e., left ventriculo-arterial coupling) in specific clinical situations. In a recent experimental study, Ea_dyn_ was found to be inversely related to left ventriculo-arterial coupling and directly to left ventricular efficiency (7). Furthermore, Ea_dyn_ was also correlated with determinants of the vascular waterfall in patients with vasoplegic syndrome (28). Therefore, Ea_dyn_ may reflect the balance between blood pressure and blood flow at the macro- and micro-circulatory levels and inform about the cardiovascular efficiency in compensating for the hemodynamic changes in response to treatment adjustments. In this regard, Ea_dyn_ should have broad clinical application scenarios in critical care medicine. A recent randomized trial established an Ea_dyn_-based hemodynamic algorithm to manage post-cardiac surgery patients with vasoplegic syndrome and showed that the algorithm was associated with a shorter duration of norepinephrine treatment and a shorter length of ICU stay (29). In a post-hoc analysis of the randomized study, the authors also found that the Ea_dyn_-based hemodynamic algorithm reduced the incidence of acute kidney injury (30). These findings are not unexpected because Ea_dyn_ may reflect left ventriculo-arterial coupling and is associated with left ventricular efficiency (7). Previous studies had documented that left ventriculo-arterial coupling was associated with oxygen consumption and tissue perfusion (31, 32); a well-matched ventriculo-arterial coupling will result in improved tissue perfusion and finally decrease postoperative complications (30). Therefore, Ea_dyn_ could also be used clinically to prevent postoperative complications, apart from informing about the excess use of vasopressor.

To the best of our knowledge, this meta-analysis is the first to systematically assess the predictive performance of Ea_dyn_ for arterial pressure changes related to norepinephrine weaning. This meta-analysis presents several major methodological strengths. We estimated the AUHSROC as the main measure of diagnostic accuracy to avoid the misinterpretation of the findings resulting from the mixed cut-off values among the included studies. In addition, we estimated the 95% CI of the optimal threshold, which may represent the “uncertain zone”, to avoid the binary constraint of a “black-or-white” decision of the ROC curve, because a single threshold seems to be inapplicable to a realistic clinical decision-making scenario. Herein, we propose a feasible decision-making algorithm to manage norepinephrine withdrawal during the de-escalation phase: (1) if the measured Ea_dyn_ is greater than 0.98, the infused dose of norepinephrine should be decreased stepwise until the measured Ea_dyn_ is within the range of 0.80–0.98; and (2) if the measured Ea_dyn_ is less than 0.80, the norepinephrine withdrawal process should be discontinued.

Nevertheless, several limitations of our study should be addressed. First, the included studies were limited and the sample sizes were small, which represent the primary limitation in this meta-analysis and hamper us from drawing a firm conclusion. Additionally, the different techniques applied to measure SVV represent a challenge to the reliability of our findings. The varied techniques measuring SVV might lead to diverse thresholds of Ea_dyn_ and between-study heterogeneities. Furthermore, the different thresholds (15% or 10%) used to define MAP responsiveness could also result in a potential heterogeneity among the included studies. However, we could not perform subgroup analyses to identify these potential effect modifiers (including the shock type, MAP responsiveness definition, norepinephrine dose, and the technique measuring SVV) due to the limited included studies. Thus, the findings should be interpreted with caution. Second, none of the included studies had a high methodological quality. The intrinsic methodological shortcomings might introduce a potential bias in the results and thereby restrict the validity and applicability of the findings. However, we conducted a sensitivity analysis by excluding the study introducing a high risk of bias, which confirmed the robustness of the findings. Lastly, we could not determine whether Ea_dyn_ has also a good diagnostic accuracy in spontaneously breathing patients. However spontaneous breathing might, theoretically, have no or at most a minor effect on the diagnostic accuracy of Ea_dyn_ because spontaneous breathing should exert the same degree of impact on the estimation of PPV and SVV.

## Conclusion

In mechanically ventilated patients with vasoplegic syndrome, the measurement of Ea_dyn_ at baseline may be a useful and reliable approach to predict arterial pressure changes in response to norepinephrine weaning. The range of 0.80–0.98 may represent the “uncertain zone” for the prediction of the MAP response to norepinephrine weaning. Given the limited included studies and participants, larger studies with high quality are warranted to validate the applicability of Ea_dyn_ in the future.

## Data Availability

The raw data supporting the conclusions of this article will be made available by the authors, without undue reservation.
