# Supplementary material for: Dynamic arterial elastance as a predictor of arterial pressure response to norepinephrine weaning in mechanically ventilated patients with vasoplegic syndrome—a systematic review and meta-analysis
Source: Front Cardiovasc Med. 2024 Feb 8;11:1350847. doi: 10.3389/fcvm.2024.1350847 (PMC10881861; doi:10.3389/fcvm.2024.1350847)
Supplement: Supplementary file 1 [file Datasheet1.docx]

**Dynamic arterial elastance as a predictor of arterial pressure response to norepinephrine weaning in mechanically ventilated patients** **with vasoplegic syndrome** **– a systematic review and meta-analysis**

**ADDITIONAL FILE 1.**

**Table of contents**

[Table S1. Detailed search strategy in each database 2](#_Toc139911751)

[Table S2. Ineligible studies and the reasons for exclusion 3](#_Toc139911752)

[Table S3. Detailed diagnostic accuracy of dynamic arterial elastance from each included study 5](#_Toc139911753)

# Table S1. Detailed search strategy in each database

| **PUBMED** (493 records) | | |
| --- | --- | --- |
| **#** | **Details** | **Records** |
| 1 | "Arterial Pressure"[Mesh] | 6952 |
| 2 | (((((((Arterial Pressure*) OR (Arterial Tension*)) OR (Blood Pressure*)) OR (Arterial Blood Pressure*)) OR (Mean Arterial Pressure*)) OR (Mean Aortic Pressure*)) OR (Radial artery pressure*)) OR (Femoral artery pressure*) | 725822 |
| 3 | #1 OR #2 | 725822 |
| 4 | (((Dynamic arterial elastance) OR (Pulse pressure variation/Stroke volume variation)) OR (PPV/SVV ratio)) OR (PPV/SVV) | 1264 |
| 5 | #3 AND #4 Filters: Humans | 493 |
| **EMBASE** (167 records) | | |
| **#** | **Details** | **Records** |
| 1 | arterial AND pressure* OR (arterial AND tension*) OR (blood AND pressure*) OR (arterial AND blood AND pressure*) OR (mean AND arterial AND pressure*) OR (radial AND artery AND pressure) OR (femoral AND artery AND pressure) | 1041668 |
| 2 | dynamic AND arterial AND elastance | 184 |
| 3 | #1 AND #2 | 167 |
| **Web of Science** (174 records) | | |
| **#** | **Details** | **Records** |
| 1 | TS=(Arterial Pressure*) OR TS=(Arterial Tension*) OR TS=(Blood Pressure*) OR TS=(Arterial Blood Pressure*) OR TS=(Mean Arterial Pressure*) | 378314 |
| 2 | TS: (Dynamic arterial elastance) OR TS: (Pulse pressure variation/Stroke volume variation) OR TS: (PPV/SVV ratio) OR TS: (PPV/SVV) | 201 |
| 3 | #1 AND #2 | 174 |
| **Cochrane Central Register of Controlled Trials** (22 records) | | |
| 1 | MeSH descriptor: [Arterial Pressure] explode all trees | 844 |
| 2 | (Arterial Pressure*):ti,ab,kw OR (Arterial Tension*):ti,ab,kw OR (Blood Pressure*):ti,ab,kw OR (Arterial Blood Pressure*):ti,ab,kw OR (Mean Arterial Pressure*):ti,ab,kw | 132130 |
| 3 | #1 OR #2 | 132130 |
| 4 | (Dynamic arterial elastance):ti,ab,kw | 35 |
| 5 | #3 AND #4 | 22 |

# Table S2. Ineligible studies and the reasons for exclusion

| **Reasons for exclusion** | **Studies** |
| --- | --- |
| Enrolling patients who did not meet the definition of vasoplegic syndrome | Vos/2013 [1] |
| Predicting arterial pressure response to fluid expansion, not norepinephrine weaning (8 studies) | Cecconi/2015 [2]; Di Tomasso/2021 [3];  Lanchon/2017 [4]; Messina/2023 [5];  Wu/2016 [6]; Seo/2015 [7];  Monge García/2011 [8];  Monge García/2014 [9];  Hikasa Y/2023 [10] |
| Predicting arterial pressure response to vasopressor infusion, not vasopressor weaning (2 studies) | Guarracino/2019 [11];  de Courson/2019 [12] |

**Reference:**

1. Vos JJ, Kalmar AF, Struys MM, Wietasch JK, Hendriks HG, Scheeren TW. Comparison of arterial pressure and plethysmographic waveform-based dynamic preload variables in assessing fluid responsiveness and dynamic arterial tone in patients undergoing major hepatic resection. Br J Anaesth. 2013;110(6):940-6.
2. Cecconi M, Monge García MI, Gracia Romero M, Mellinghoff J, Caliandro F, Grounds RM, et al. The use of pulse pressure variation and stroke volume variation in spontaneously breathing patients to assess dynamic arterial elastance and to predict arterial pressure response to fluid administration. Anesth Analg. 2015;120(1):76-84.
3. Di Tomasso N, Lerose CC, Licheri M, Castro LEA, Tamà S, Vitiello C, et al. Dynamic arterial elastance measured with pressure recording analytical method, and mean arterial pressure responsiveness in hypotensive preload dependent patients undergoing cardiac surgery: A prospective cohort study. Eur J Anaesthesiol. 2021;38(4):402-410.
4. Lanchon R, Nouette-Gaulain K, Stecken L, Sesay M, Lefrant JY, Biais M. Dynamic arterial elastance obtained using arterial signal does not predict an increase in arterial pressure after a volume expansion in the operating room. Anaesth Crit Care Pain Med. 2017;36(6):377-382.
5. Messina A, Colombo D, Lionetti G, Calabrò L, Negri K, Robba C, et al. Pressure response to fluid challenge administration in hypotensive surgical patients: a post-hoc pharmacodynamic analysis of five datasets. J Clin Monit Comput. 2023;37(2):449-459.
6. Wu CY, Cheng YJ, Liu YJ, Wu TT, Chien CT, Chan KC; NTUH Center of Microcirculation Medical Research (NCMMR). Predicting stroke volume and arterial pressure fluid responsiveness in liver cirrhosis patients using dynamic preload variables: A prospective study of diagnostic accuracy. Eur J Anaesthesiol. 2016;33(9):645-52.
7. Seo H, Kong YG, Jin SJ, Chin JH, Kim HY, Lee YK, et al. Dynamic Arterial Elastance in Predicting Arterial Pressure Increase After Fluid Challenge During Robot-Assisted Laparoscopic Prostatectomy: A Prospective Observational Study. Medicine (Baltimore). 2015;94(41): e1794.
8. Monge García MI, Gil Cano A, Gracia Romero M. Dynamic arterial elastance to predict arterial pressure response to volume loading in preload-dependent patients. Crit Care. 2011;15(1): R15.
9. Monge García MI, Romero MG, Cano AG, Aya HD, Rhodes A, Grounds RM, et al. Dynamic arterial elastance as a predictor of arterial pressure response to fluid administration: a validation study. Crit Care. 2014;18(6):626.
10. Hikasa Y, Suzuki S, Tanabe S, Noma K, Shirakawa Y, Fujiwara T, et al. Stroke volume variation and dynamic arterial elastance predict fluid responsiveness even in thoracoscopic esophagectomy: a prospective observational study. J Anesth. 2023;37(6):930-937.
11. Guarracino F, Bertini P, Pinsky MR. Cardiovascular determinants of resuscitation from sepsis and septic shock. Crit Care. 2019;23(1):118.
12. de Courson H, Boyer P, Grobost R, Lanchon R, Sesay M, Nouette-Gaulain K, et al. Changes in dynamic arterial elastance induced by volume expansion and vasopressor in the operating room: a prospective bicentre study. Ann Intensive Care. 2019;9(1):117.

| Study No. | | Author/year | AUROC | Cut-off value | Sensitivity (%) | Specificity (%) | True positive | False positive | False negative | True negative |
| --- | --- | --- | --- | --- | --- | --- | --- | --- | --- | --- |
| 1 | Guinot/2015 | | 0.87 | 0.94 | 100 | 68 | 13 | 7 | 0 | 15 |
| 2 | Liang/2017 | | 0.85 | 0.97 | 100 | 73.7 | 13 | 5 | 0 | 14 |
| 3 | Bar/2018 | | 0.84 | 0.90 | 91 | 80 | 10 | 5 | 1 | 19 |
| 4 | Nguyen/2021 | | 0.86 | 0.80 | 92 | 74 | 11 | 7 | 1 | 20 |
| 5 | Persona/2022 | | 0.84 | 0.84 | 89 | 71 | 16 | 7 | 2 | 17 |

# Table S3. Detailed diagnostic accuracy of dynamic arterial elastance from each included study
